# Structural studies of Parvoviridae capsid assembly and evolution: implications for novel AAV vector design

**DOI:** 10.3389/frai.2025.1559461

**Published:** 2025-04-02

**Authors:** Heather A. Noriega, Qizhao Wang, Daozhan Yu, Xiang Simon Wang

**Affiliations:** ^1^Department of Pharmaceutical Sciences, Artificial Intelligence and Drug Discovery Core Laboratory for District of Columbia Center for AIDS Research (DC CFAR), College of Pharmacy, Howard University, Washington, DC, United States; ^2^AAVnerGene Inc., Rockville, MD, United States

**Keywords:** adeno-associated virus, Parvoviridae, structure-guided design, directed evolution, AI/ML, vector design

## Abstract

Adeno-associated virus (AAV) vectors have emerged as powerful tools in gene therapy, potentially treating various genetic disorders. Engineering the AAV capsids through computational methods enables the customization of these vectors to enhance their effectiveness and safety. This engineering allows for the development of gene therapies that are not only more efficient but also personalized to unique genetic profiles. When developing, it is essential to understand the structural biology and the vast techniques used to guide vector designs. This review covers the fundamental biology of the Parvoviridae capsids, focusing on modern structural study techniques, including (a) Cryo-electron microscopy and X-ray Crystallography studies and (b) Comparative analysis of capsid structures across different Parvoviridae species. Along with the structure and evolution of the Parvoviridae capsids, computational methods have provided significant insights into the design of novel AAV vector techniques, which include (a) Structure-guided design of AAV capsids with improved properties, (b) Directed Evolution of AAV capsids for specific applications, and (c) Computational prediction of AAV capsid-receptor interactions. Further discussion addressed the ongoing challenges in the AAV vector design and proposed future directions for exploring enhanced computational tools, such as artificial intelligence/machine learning and deep learning.

## Introduction

**Figure 1:**
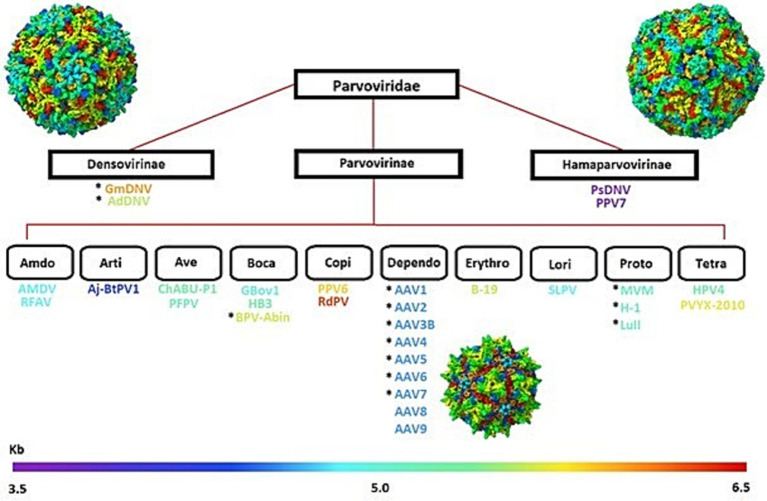
Classificational diagram of the Parvoviridae family, illustrating the three main subfamilies- Densovirinae, Parvovirinae, and Hamaparvovirinae. Subfamily subdivisions highlight diverse members, such as the Dependoparvovirus (AAV1-AAV9), organized by genome size (kb), represented by the gradient bar at the bottom. Structural representations of capsids from each subfamily are included for visual reference.

The Parvoviridae family includes a group of small, non-enveloped, single-stranded DNA packaging viruses with genomes of ~4–6 kb that infect a wide range of animals. The family for many years consisted of two families until, in recent years, three categorical subfamilies emerged: Parvovirinae, Densovirinae, and Hamaparvovirinae (Penzes et al., 2020b), with additional subfamilies to each category with a focus on Parvovirinae, as shown in Figure 1. The capsid in this family is considered a T = 1 icosahedron, which is composed of 60 viral protein monomers. The assembly of these viral proteins forms by the alternate splicing of the same messenger RNA (mRNA), which in some families include viral protein 1 (VP1) and viral protein 2 (VP2), with a general ratio of 1:10 (Mietzsch et al., 2019). A third structural protein, VP3, has been identified in some viruses, including the AAV (Mietzsch et al., 2019). For the AAV family known as Dependoparvovirus, the three proteins VP1, VP2, and VP3 tend to show a general capsid ratio of 1:1:10 but have been observed to vary from virus to virus (Worner et al., 2021; Emmanuel et al., 2021). Members of the Densovirinae are also known to express multiple structural proteins, with four VPs (VP1-VP4) being the most common and various ratios (Penzes and Kaelber, 2022). However, further research needs to be conducted as there is minimal information on other viruses besides the Galleria Mellonella Densovirus (GmDV) and Archeta Domesticus Densovirus (AdDV).

The capsid proteins all contain a central eight-stranded, anti-parallel *β*-barrel motif, and the four extended loops link the strands of the β-barrel. The HI loop is prominent on the AAV capsid and extends from each VP subunit, overlapping the neighboring fivefold VP. The EF loop is located between the β barrel motif (DiPrimio et al., 2008). At the center of the peaks is part of the GH loop sandwiched by two other subloops (Xie et al., 2002). These loops form most of the outer surface of the virus particle and are responsible for their receptor binding, antigenic properties, and environmental stability (Emmanuel et al., 2021; Casanovas, 2013). This assembly coordinates at 12 5-fold, 20 3-fold, and 30 2-fold axes. The surface of the capsid feature includes depressions at each icosahedral 2-fold axis, with elevated protrusions surrounding the 3-fold axis and raised cylindrical projections encircling each 5-fold vertex surrounded by extensive canyon-like depressions. Surface features like this are prevalent in AAV; however, relative elevations vary between family members and other subfamilies (Cotmore et al., 2019; Penzes et al., 2020b). These are significant findings that have been used over the years to advance our understanding of the structure base of the Parvoviridae using Cryo-Electron microscopy (cryo-EM) and X-ray crystallography research.

Understanding these structures is important, as they are closely related to the assembly. Capsid assembly is a tightly regulated process that ensures proper encapsidation of the viral genome, stability, and infectivity. During assembly, capsid proteins interact through specific binding sites, facilitating the formation of the icosahedral shell. This structural assembly protects the viral genome and enables the capsid to undergo conformational changes necessary for host cell entry and subsequent gene delivery (Large et al., 2021). Nevertheless, understanding fully these structural insights of the assemblies and mechanical properties has been challenging, time-consuming, and expensive (Mateu, 2012; Cotmore and Tattersall, 2014; Hafenstein et al., 2009; Hadden et al., 2018; Leisi et al., 2020; Zhang Y. et al., 2019; Roos et al., 2007).

Vice versa, the attraction to Parvoviridae is the simplicity of their genomes and capsids, which makes them excellent models for studying fundamental aspects of virology. Also, many members of the Parvoviridae family are pathogenic to animals, including humans, making them important targets for therapeutic intervention. Diseases caused by parvoviruses, such as canine parvovirus in dogs and parvovirus B19 in humans, emphasize the necessity for detailed structural and functional analyses to inform vaccine and antiviral drug development (Lopez-Astacio et al., 2023).

More specifically, AAVs, part of the Parvovirinae subfamily, have gained significant attention due to their utility in gene therapy (Hadi et al., 2024; Wang et al., 2024). AAVs are non-pathogenic and have a high safety profile, making them ideal vectors for delivering therapeutic genes to target cells (Weller et al., 2010; Nam et al., 2011; Wang et al., 2024). This facilitates advancements in treating genetic disorders (Monahan et al., 2010). Thus, studying Parvoviridae shows both pathogenic viruses and therapeutic vectors, like AAVs, are fundamental to developing effective treatments and preventive measures (Denby et al., 2005). A major contribution to this effort within the last few years has been using computational-aided drug design and artificial intelligence (AI), which have had some breakthroughs in hybrid variants using structure-guided methods, directed evolution techniques, and machine learning (ML).

This review synthesizes findings from articles published over 30 years (1996–2024), focusing on a two-part component. It explores the fundamental biology of the Parvoviridae capsids, the use and examination of Cryo-electron microscopy and X-ray crystallography studies, and a comparative analysis of capsid structures across different Parvoviridae species. Additionally, it will address how computational methods have contributed to the development of AAV vector techniques. This includes designing AAV capsids with improved properties, using structure-guided evolution and directed evolution to create AAV capsids for specific purposes, and predicting AAV capsid-receptor interactions. Then, we will continue to discuss ongoing challenges in the AAV vector design and suggest directions for future research using advanced computational tools, AI/ML, and DL. While previous reviews have addressed the structural biology and evolution of Parvoviridae capsids, this review integrates AI-driven approaches in AAV vector engineering. By incorporating computational insights alongside traditional structural studies, we provide a comprehensive perspective on how these emerging technologies are shaping the field. This highlights the historical advancements and new methodologies that enhance the capsid modifications.

### Structural insights and comparative studies of Parvoviridae capsids

Comparative studies within the Parvoviridae family have been shown to provide insights into determinants of assembly, stability, transduction, and tissue tropism, such as the ability to cross the blood–brain barrier, retinal, heart, and other areas of interest (Mietzsch et al., 2020a). Structural bioinformatics resources, such as VIPERdb and Protein Data Bank, (Berman et al., 2003; Berman et al., 2000) provide Cryo-EM and X-ray crystallography data, which optimize capsid structures and evaluate their efficacy (Hull et al., 2022; Callaway et al., 2017; Mietzsch et al., 2019). Studies focusing on adaptations and evolutionary origins, particularly those of the Parvoviridae family, have demonstrated potential clinical applications and enhanced transduction efficiency (Hildebrandt et al., 2020; Gonzalez et al., 2022).

For example, studies have demonstrated that AAV9 utilizes the terminal N-linked galactose as its primary receptor, which enhances its ability to transduce neurons. This study has demonstrated that AAV9 shows higher efficiency in crossing the blood–brain barrier compared to AAV2, leading to a more effective delivery to the CNS (Merkel et al., 2016). Additionally, another study indicates that AAV8 achieves higher transduction efficiency in mouse liver than AAV2 or AAV5. However, while highly effective in mouse models, AAV8 is less effective in human and non-human primate hepatocytes, driven by the structural differences between serotypes. Further, it shows the importance of understanding the structural aspects and environmental payloads in the Cryo-Em and X-ray studies (Zhao et al., 2024).

Cryo-EM and X-ray crystallography maps have helped explain the atomic structures of various Parvoviridae capsids. For instance, the structure of AAV2 was resolved at 1.86 Å resolution using cryo-EM, providing insights into the capsid organization, including backbone tracing with well-defined carbonyls, the explicit structure of most side chains, ordered solvent, and even traces of hydrogen atoms. These are all important to offer information about potential interaction sites with host cell receptors (Xie et al., 2002). Similarly, the structures of AAV1-13, AAVrh.10, AAVrh.39, and AAVrh.8 have been analyzed, revealing critical structural differences that influence their tropism and transduction efficiency due to pH levels (Mietzsch et al., 2020a; Nam et al., 2011; DiMattia et al., 2012). Comparative structural analyses across different Parvoviridae subfamilies and the AAV serotypes have identified conserved regions for capsid stability and variable regions (VRs) that can be engineered to improve the delivery. A study by Rayaprolu et al. (2013) compared the physical properties of AAV1, AAV2, AAV5, and AAV8 capsids that influence thermodynamic stability. Their thermal stability measurements using differential scanning fluorimetry, differential scanning calorimetry, and electron microscopy showed that capsid melting temperatures differed more than 20° C between the least and most stable serotypes, AAV2 and AAV5. The study found AAV5 was most stable, with a melting temperature of approximately 5°C, 17°C, and 20°C higher than AAV1, AAV8, and AAV2. Interestingly, the common VP ratio of 1:1:10 for VP1, VP2, and VP3 varies widely among studies depending on serotype and assembly conditions. For instance, Xie et al. (2002) Observed ratios closer to 1:1:10 in some lab preparations for AAV2 while other studies reported ratios ranging from 1:1:8 to 1:1:10 depending on the virus and preparation (Chapman and McKenna, 2005). Another study showed that higher VP3 presence is often associated with greater capsid stability, which is attributed to increased hydrophobic interactions that enhance capsid rigidity. This structural rigidity reduces susceptibility to environmental stressors, such as temperature or pH variations. Limited proteolysis and peptide mass mapping of intact particles were used to investigate the capsid protein dynamics. Active hot spots mapped to the region surrounding a 3-fold axis of symmetry for all serotypes. Surface-exposed hydrophobic residues near the 3-fold axis promote initial host binding, while transient VP1 unique regions (VP1u) exposure, seen in some capsids, enhances interaction stability. The phospholipase-2 (PLA-2) domain in VP1u relies on transients’ polarity shifts that allow it to function with varying membrane environments (Rayaprolu et al., 2013).

Studies focusing on the capsids have demonstrated comprehensive applications. These structural data allowed researchers to design hybrid AAV vectors with enhanced properties, such as improved stability and immune evasion (Worner et al., 2021; Emmanuel et al., 2021). For example, the study published in Nature in 2022 described a cross-species evolution approach that yielded an AAV variant, AAV.cc47, with improved transduction in mouse liver, CNS, cardiac, and skeletal muscle tissue compared to AAV9. Structural adjustments in AAV.cc47, such as shifts toward hydrophobic or neutral side chains in the VR-IV regions (452-GVSLGGG-458), correlate with its enhanced stability and delivery capabilities. These changes have led to higher genome editing efficiencies in the mouse heart and skeletal muscle when using CRISPR/Cas9 cassettes packaged in AAV.cc47 compared to AAV9 (Gonzalez et al., 2022).

Studies on other subfamilies, Densovirinae and Hamaparvovirinae, have also provided understanding. For instance, the atomic structure of Densovirinae capsids has been determined, including GmDV at 3.7 Å resolution and *Bombyx Mori* Densovirus (BmDV) at 3.1 Å resolution. These structures showed essential features, including symmetry, 60-unit monomers, and the conserved eight-stranded *β*-barrel motif that forms the cores of the capsid protein, which were similar to the other subfamilies. However, studies have shown a diverse VP ratio among the Densovirinae members; for example, the GmDV capsid assembles with VP1 to VP4 in a 1:9:9:41 ratio, while the AdDV incorporates its VPs in a 1:1:18:30 ratio showing variability even within the subfamily (Penzes and Kaelber, 2022). Additionally, Zophobas Morio Beetle Densovirus (ZmBMV) shows approximately equal incorporations of VP4 and VP5 as major structural proteins, suggesting species-specific assembly adaptations (Penzes and Kaelber, 2022). Similarly, structural studies on Hamaparvovirinae, the capsid structure of *Penaeus Stylirostris* Densovirus (PstDV) at 2.5 Å resolution, have contributed capsid properties and their interactions with even smaller capsids <4 kb. PstDV has been shown to incorporate only a single VP protein-VP1, making it distinct in recent years to be a part of the new subfamily- Hamaparvovirinae, showing smaller capsids within the scope of Parvoviridae (Kryshtafovych et al., 2021; Louten, 2016; Cotmore and Tattersall, 2014). An overall Table 1 has been provided to show what structural Parvoviridae studies have contributed insights and the importance to the Cyro-EM and X-ray crystallography maps that have provided information for structural conserved areas, hybrid variants, stability, receptor interactions, and tissue tropism. These structural studies have progressed into more advanced methods, which we will discuss further in relation to traditional computational methods.

**Table 1:** Shows structural Parvoviridae studies that have contributed insights to the Cyro-EM and X-ray crystallography maps and to the field.

Focus of study	Year	Impact on field	Methods	References
Structural analysis of a mutation in Canine Parvovirus which controls antigenicity and host range	1996	Provides insights into how viral mutations can alter host specificity and immune response evasion.	X-ray crystallography	Llamas-Saiz et al. (1996)
The structure of an insect parvovirus (Galleria Mellonella densovirus) at 3.7 Å resolution	1998	It provides insights into insect parvovirus structure, aiding in understanding virus-host interactions in insects.	X-ray crystallography, Cryo-Em	Simpson et al. (1998)
Host range and variability of calcium binding by surface loops in the capsids of canine and feline parvoviruses	2000	Enhances understanding of capsid stability and infection mechanisms in parvoviruses.	X-ray crystallography	Simpson et al. (2000)
The atomic structure of adeno-associated virus (AAV-2), a vector for human gene therapy	2002	Pioneered structural understanding of AAV, aiding in its use for gene therapy.	Cryo-EM, X-ray crystallography	Xie et al. (2002)
The structure of porcine parvovirus: comparison with related viruses	2002	Enhances understanding of parvovirus structure using a comparison method of different serotypes	Cryo-EM	Simpson et al. (2002)
Structures of host range-controlling regions of the capsids of Canine and Feline Parvoviruses and Mutants	2003	Enhances knowledge of parvovirus host specificity and can guide vaccine and therapy development.	X-ray crystallography	Govindasamy et al. (2003)
Structural determinants of tissue tropism and *in vivo* pathogenicity for the Parvovirus minute virus of mice	2005	Provides insights into viral infection mechanisms, aiding in the development of tissue-specific vectors.	Structural modeling	Kontou et al. (2005)
Structurally mapping the diverse phenotype of adeno-associated virus serotype 4	2006	Helps to understand AAV4’s distinct biological properties and guide vector development.	Cryo-EM	Govindasamy et al. (2006)
Structure of adeno-associated virus serotype 8, a gene therapy vector	2007	Advances the understanding of AAV8’s structural properties, aiding in its use for gene therapy.	Cryo-EM, structural modeling	Nam et al. (2007)
Visualization of the externalized VP2 N termini of infectious human parvovirus B19	2008	Provides insights into the assembly and infection mechanisms of human parvovirus B19.	Cryo-EM, mass spectrometry	Kaufmann et al. (2008)
The structure of adeno-associated virus serotype 3B (AAV-3B): insights into receptor binding and immune evasion	2010	Enhances understanding of AAV-3B interactions with the immune system and potential receptors.	Cryo-EM	Lerch et al. (2010)
Structural characterization of the dual glycan binding adeno-associated virus serotype 6	2010	It provides crucial insights into AAV6’s cell entry and potential for targeting specific tissues.	Cryo-EM	Ng et al. (2010)
Human Bocavirus capsid structure: insights into the structural repertoire of the *Parvoviridae*	2010	Expands the structural repertoire known within the Parvoviridae family, aiding in therapeutic design.	X-ray crystallography, Cryo-EM	Gurda et al. (2010)
Structure of *Penaeus Stylirostris* Densovirus, a shrimp pathogen	2010	Enhances the understanding of viral diseases in shrimp and the structural principles in the Densovirus family	Cryo-EM, comparative structural modeling	Kaufmann et al. (2010)
Structure–function analysis of receptor-binding in adeno-associated virus serotype 6 (AAV-6)	2011	Enhances understanding of AAV-6 cell entry mechanisms and guides vector development for receptor binding.	Cryo-EM	Xie et al. (2011)
Structural studies of adeno-associated serotype 8 capsid transitions associated with endosomal trafficking	2011	Provides insight into the cellular processing of AAV8, enhancing vector design.	Cryo-EM	Nam et al. (2011)
Structure of a packaging-defective mutant of minute virus of mice indicates that the genome is packaged via a pore at a 5-fold axis	2011	Enhances understanding of the viral assembly process and genome packaging.	Cryo-EM	Plevka et al. (2011)
Structure of adeno-associated virus-2 in complex with neutralizing monoclonal antibody A20	2012	Enhanced understanding of viral immune evasion and AAV-2 capsid-antibody interactions.	Cryo-EM	McCraw et al. (2012)
Structural Insight into the unique properties of adeno-associated virus serotype 9	2012	Advances the understanding of AAV9, aiding in its utilization for targeting	Cryo-EM	DiMattia et al. (2012)
Structural insights into adeno-associated virus serotype 5	2013	Contributes to the targeted design of AAV5-based vectors.	X-ray crystallography, structural comparison	Govindasamy et al. (2013)
Structural characterization of H-1 Parvovirus: comparison of infectious Virions to empty capsids	2013	Enhances knowledge of H-1 parvovirus assembly and infection mechanisms.	Cryo-EM, structural modeling	Halder et al. (2013)
The structure and host entry of an invertebrate Parvovirus	2013	Expands knowledge of parvovirus biology across different species and environments.	Cryo-EM	Meng et al. (2013)
Structure of neurotropic adeno-associated virus AAVrh.8	2015	Enhances understanding of AAVrh.8’s mechanisms for nervous system targeting and infection.	Cryo-EM	Halder et al. (2015)
Structure of an enteric pathogen, Bovine Parvovirus	2015	Aids in the understanding of enteric pathogens in the Bovine Parvovirus	Cryo-EM	Kailasan et al. (2015)
Global displacement of Canine Parvovirus by a host-adapted variant: structural comparison between pandemic viruses with distinct host ranges	2015	Highlights the role of structural changes in viral capsids in host adaptation and global virus displacement using the Canine Parvovirus 2a	Cryo-EM	Organtini et al. (2015)
Cryo-electron microscopy reconstruction and stability studies of the wild type and the R432A variant of adeno-associated virus type 2 reveal that capsid structural stability is a major factor in genome packaging	2016	Highlights the importance of capsid stability in AAV’s genome packaging efficiency using AAV2 wild type and variants	Cryo-EM	Drouin et al. (2016)
Near-atomic resolution structure of a highly neutralizing fab bound to Canine Parvovirus	2016	Enhances understanding of neutralization mechanisms and informs vaccine and therapeutic design using the Canine Parvovirus	Cryo-EM	Organtini et al. (2016)
Structural insights into human Bocaparvoviruses	2017	Enhances the understanding of Bocaparvovirus structure.	Cryo-EM	Mietzsch et al. (2017)
Structural basis for biologically relevant mechanical stiffening of a virus capsid by cavity-creating or space-filling mutations	2017	Provides structural insights into how mutations can enhance viral capsid rigidity.	Cryo-EM	Guerra et al. (2017)
Atomic resolution structure of the oncolytic parvovirus LuIII by electron microscopy and 3D image reconstruction	2017	Aids in understanding LuIII’s structure for potential therapeutic applications in oncolytic therapy.	Cryo-EM	Pittman et al. (2017)
Atomic structure of a rationally engineered gene delivery vector, AAV2.5	2018	Enhances the design and development of AAV2.5 vectors.	Cryo-EM	Burg et al. (2018)
Atomic resolution structures of human Bufaviruses determined by Cryo-electron microscopy	2018	Contributes to the understanding of Bufavirus structure and potential human pathogenicity.	Cryo-EM	Iiyas et al. (2018)
Divergent engagements with cellular receptor AAVR	2019	Enhanced understanding of AAV-receptor interactions for improving vector targeting and design.	Cryo-EM	Zhang et al. (2019b)
Adeno-associated virus 2 bound to its cellular receptor AAVR	2019	Enhanced understanding of AAV2’s cellular entry.	Cryo-EM	Zhang et al. (2019a)
Structure of the gene therapy vector, adeno-associated virus with its cell receptor, AVVR	2019	Provides insights into the structure of the AAVR	Cryo-EM	Meyer et al. (2019)
Transferrin receptor binds virus capsid with dynamic motion	2019	Provides insights into virus-receptor interactions using the Canine Parvovirus 2 and 2a variant	Cryo-EM	Lee et al. (2019)
The structure of an AAV5-AAVR complex at 2.5 Å resolution: implications for cellular entry and immune neutralization of AAV gene therapy vectors	2020	Provides information on AAV5’s cellular entry and immune evasion.	Cryo-EM	Silveria et al. (2020)
Comparative analysis of the capsid structures of AAVrh.10, AAVrh.39, and AAV8	2020	Enhances understanding of structural differences affecting tissue tropism and immune escape among AAV variants	Cryo-EM, tissue tropism mapping	Mietzsch et al. (2020a)
Methods matter: standard production platforms for recombinant AAV produce chemically and functionally distinct vectors	2020	Highlights the importance of production methods on AAV vector characteristics and efficacy.	Cryo-EM	Rumachik et al. (2020)
Structural characterization of a bat Adeno-associated virus capsid	2020	Expands the diversity and understanding of AAV bat capsids, potentially aiding in vector development.	Cryo-EM	Mietzsch et al. (2020b)
Structural characterization of Cuta- and Tusavirus: insight into Protoparvoviruses capsid morphology	2020	Provides a structural understanding of the diverse aspects of Protoparvoviruses.	Cryo-EM	Mietzsch et al. (2020c)
Molecular biology and structure of a novel penaeid shrimp Densovirus elucidate convergent parvoviral host capsid evolution	2020	Provides biology and structure of shrimp Densovirus convergent evolution across different hosts to help the understanding of virus-host interactions.	Cryo-EM	Penzes et al. (2020a)
Comparative structural, biophysical, and receptor binding study of true type and wild type AAV2	2021	Enhances understanding of AAV2 receptor interactions and implications for tissue tropism and immune response.	Cryo-EM	Bennett et al. (2021)
Completion of the AAV structural atlas: serotype capsid structures reveals clade-specific features	2021	Provides a comprehensive understanding of AAV structural variations for gene therapy applications.	Cryo-EM, structural development	Mietzsch et al. (2021)
Receptor switching in newly evolved Adeno-associated viruses	2021	Advances understanding of AAV evolution and receptor interactions.	Cryo-EM	Havlik et al. (2021)
Adeno-associated Virus 9 structural rearrangements induced by endosomal trafficking pH and glycan attachment	2021	Enhances understanding of AAV9’s cell entry mechanism, influencing vector development.	Cryo-EM	Penzes et al. (2021)
Context-specific function of the engineered peptide domain of PHP.B	2021	Aids in understanding and optimizing the tissue-specific targeting of AAV.PHP.B vectors.	Cryo-EM	Martino et al. (2021)
Structural study of Aavrh.10 receptor and antibody interactions	2021	Advances understanding of AAVrh.10’s cellular entry and immune evasion.	Cryo-EM	Mietzsch et al. (2021)
Characterization of the GboV1 capsid and its antibody interactions	2021	Enhances understanding of GBoV1’s immune evasion mechanisms and capsid antigenicity.	Cryo-EM	Yu et al. (2021)
pH-induced conformational changes of human Bocavirus capsids	2021	Provides insights into the environmental triggers affecting Bocavirus stability and infectivity.	Cryo-EM	Luo et al. (2021)
High-resolution asymmetric structure of a Fab-virus complex reveals overlap with the receptor binding site	2021	Provides insights into virus-antibody interactions with the Canine Parvovirus and implications for vaccine and therapy design.	Cryo-EM	Goetschius et al. (2021)
Structural characterization of an envelope-associated adeno-associated virus type 2 capsid	2022	Provides insights into the enveloped-associated structural biology of AAV2	Cryo-EM	Hull et al. (2022)
Structural basis for the neurotropic AAV9 and the engineered AAV PHP.eB recognition with cellular receptors	2022	Improves understanding of vector-receptor interactions using the AAV9 and AAV.PHP.eB variant	Cryo-EM	Xu et al. (2022)
Cross-species permissivity: structure of a goat adeno-associated virus and its complex with the human receptor AAVR	2022	Expands the understanding of AAVR cross-species infectivity and receptor binding mechanisms.	Cryo-EM	Large et al. (2022)
Structural basis of receptor usage by the engineered capsid AAV-PHP.eB	2022	Contributes to the development of AAV.PHP.eB vectors for targeted therapy in neurological disorders.	Cryo-EM	Jang et al. (2022)
Capsid structure of Aleutian mink disease virus and human Parvovirus 4: new faces in the parvovirus family portrait	2022	Enhances the understanding of the Aleutian Mink Disease Virus structure and comparison between the HP4, belonging to newer subclasses: Amdoparvovirus and Tetraparvovirus in Parvovirinae	Cryo-EM	Lakshmanan et al. (2022)
Characterization of the serpentine Adeno-Associated virus capsid structure: receptor interactions and antigenicity	2023	Provides new insights into SAAV structure and receptor interactions.	Cryo-EM	Lopez-Astacio et al. (2023)

### Traditional computational techniques for AAV vector design

Through the years, structural studies have been shown to have value in the information from the studies that provide a direction for gene therapy. However, it was time-consuming and expensive. The evolving field has since started using traditional computational techniques, together with structure-guided evolution and directed evolution, which have led to improvements in the AAV capsid functionality (Havlik et al., 2020; Gonzalez et al., 2023; Tabebordbar et al., 2021; Han et al., 2023). Advances in computational methods, including molecular dynamics simulations (MDS) (Drouin and Agbandje-McKenna, 2013), and structural bioinformatics (Large et al., 2022; Mietzsch et al., 2022; Becker et al., 2022; Jang et al., 2022; Lakshmanan et al., 2022) have further guided the development of these vectors.

Molecular dynamics simulations help predict capsid stability and interactions (Lee et al., 2018). These simulations allow for assessing how different capsid mutations might affect the vector’s structural integrity and functionality. For instance, a recent molecular dynamics study showed that mutations in the VP1 unique region of AAV2 can alter thermal stability, with important implications for vector packaging and storage (Pietersz et al., 2021).

Combining MDS, structural bioinformatics, and structure-guided evolution techniques have enhanced properties in the AAV variants. For example, researchers developed AAV-PHP.B, a variant that shows an increase in the central nervous system transduction compared to AAV9, opening new possibilities for treating neurodegenerative disease. The PHP.B peptide was inserted between amino acids 588 and 589, thus enhancing its ability to cross the blood–brain barrier (Gonzalez et al., 2023; Jang et al., 2022). Additionally, computational predictions of capsid-receptor interactions have provided valuable insights into tissue tropism, facilitating the targeted delivery of AAV vectors to many tissues and diseases or disorders (Guo et al., 2024). For instance, Anc80L65 was developed using this approach, showing potential for gene therapy in the inner ear and eye (Lu et al., 2022).

Another combination using MDS and structural bioinformatics, along with directed evolution strategies, has led to the development of AAV vectors with significantly enhanced properties. For example, the rational design of AAV2.5 has demonstrated how structural modifications can improve vector performance. The structure of AAV 2.5 was determined by 2.78 Å using cryo-EM, confirming that rational design achieved the desired capsid surface properties (Lee et al., 2018). Additionally, directed evolution has resulted in AAV variants with specific tissue-targeting capabilities. For instance, a computer-aided directed evolution system paradigm was used to design a gene mutation library through ML, which we will discuss more in the next section. This led to the development of AAV2 variants (AAV2.A1 and AAV2.A2) with higher transduction efficiency than wild-type AAV2 (Han et al., 2023).

The structural studies from Cryo-EM and X-ray crystallography have provided us with a beginning marker for progression in the field. The High-resolution structures, such as the 1.56 Å resolution structure of the AAV-DJ, represent one of the most accurately interpreted AAV capsid models to date (Xie et al., 2020). These detailed structures enable the annotation of water networks required for capsid stability, protonation states, important residues, and accurate receptor and antibody interactions. Such insights are essential for rational design but have also aided in conserving time to run MDSs on more promising variants and to create more advanced methods such as directed and structure-guided evolution.

These advanced techniques have even allowed researchers to target specific regions in the AAV capsid for modification. The VRs exposed on the surface (VR-I through VR-VIII) have been the focus of many engineering efforts. The ability to modify and create variants with enhanced tissue specificity, reduced immunogenicity, and improved transduction efficiency has shown promise. However, it still has challenges. In one of the studies above, ML was used to assist in producing a mutational library, but it has not gone unnoticed. It is used in this challenge and will be further discussed with other challenges and proposed future directions in Artificial intelligence/machine learning (AI/ML).

### AI/ML addresses AAVs’ challenges and poses future directions

Immunogenicity is a fundamental hurdle, as the immune system can recognize and neutralize the viral capsids, reducing the therapy’s efficacy. Addressing the root causes of these immune responses is essential to enhancing the safety and effectiveness of AAV vectors (Hamilton and Wright, 2021). Another significant challenge is achieving precise targeting of AAV vectors to specific tissues or cell types. Enhancing tissue tropism and specificity remains critical for effective gene therapy (Guo et al., 2024). It is also essential to ensure the stability of the AAV capsid under physiological conditions and maintain its functionality for efficient gene delivery.

AI/ML and DL have emerged as powerful tools in analyzing large datasets of capsid sequences and structures, identifying patterns that may not be immediately apparent through traditional methods (Marques et al., 2021; Chen and Siu, 2020). For instance, a state-of-the-art ML model has been developed to predict the fitness of AAV capsid mutants based on their amino acid sequences, achieving high prediction accuracy and enabling rapid analysis of more sequences than traditional assays (Wu et al., 2024). This approach helps filter out non-viable sequences, reducing cost and improving manufacturability. A 2023 study by Campos et al. (2023) utilized ML to predict the AAV capsid properties, resulting in AAV.CAP-Mac is a capsid with higher transduction efficiency in the blood–brain barrier than other AAV serotypes.

Another notable breakthrough with AI/ML is the accurate prediction of protein structures. Computational prediction of protein structures has been a central challenge in molecular biology for over 50 years. The state-of-the-art AlphaFold2, a DL-based model developed by DeepMind, has addressed this problem by achieving near-experimental accuracy in protein-structure prediction, advancing the field (Capponi and Wang, 2024). AlphaFold2 was trained on millions of experimentally solved protein structures, allowing it to jointly embed multiple sequence alignments (MSA) and pairwise features, a new output representation, and associated loss that enables accurate end-to-end protein structure predictions. It can predict 3D coordinates of all heavy atoms for a given protein using the primary amino acid sequence and aligned sequences of homologs as inputs and visualized on an atomic scale (Jumper et al., 2021). This network has surpassed previous computational methods such as homology modeling and molecular dynamic simulations. AlphaFold2 has transformed protein engineering, drug discovery, and viral vector optimization by enhancing previously intractable protein structures. It has demonstrated conformational changes upon mutations and facilitated *de novo* protein design (Kolesnik et al., 2024). The model’s impact extends beyond the single-chain proteins, as AlphaFold-Multimer, an extension of the original framework, has improved the accuracy of protein–protein interaction predictions, essential for studying AAV capsid-host interactions. In 2024, by integrating this network Lee (2024) demonstrated how multi-chain AlphaFold2 structures were integrated into protein language models to enhance AAV capsid engineering, supporting the design of the functionally optimized variants. Another 2024 study utilized AlphaFold2-based modeling to develop receptor-independent AAV vectors, which no longer rely on naturally occurring receptor interactions for cell entry. This approach expands the potential applications for tissues where receptor availability is limited (Ding et al., 2023). In 2025, AlphaFold2-based modeling was used to optimize AAV Vectors for hemophilia A gene therapy, facilitating the engineering variants with improved factor VIII (F8) expression (Zhao et al., 2025). Even more recently, AlphaFold3, a diffusion-based DL model that builds upon the success of AlphaFold2, has been introduced. Unlike its predecessor, which primarily focused on single-protein structures, AlphaFold3 enables joint structural prediction of proteins, nucleic acids, small molecules, ions, and modified residues. Thus, this represents a major step forward in modeling complex biomolecular interactions (Abramson et al., 2024).

As shown, DL techniques can further enhance the structural bioinformatics of AAV vectors, providing a more atomic visual, structural, and physiological aspect, predicting interactions between AAV capsids and cellular receptors to improve targeting efficiency and specificity (Kumar and Srivastava, 2024; Chakraborty et al., 2024). Other studies have also focused on immunogenicity and packaging issues using AI/ML. In 2022, research applied DL models to predict AAV capsid immunogenicity to help facilitate the design of vectors with reduced immune response (Han et al., 2023). Similarly, work by Deverman and Eid developed an ML-driven approach to designing multifunctional AAV libraries. Traditional AAV design relied on creating large capsid libraries and selection rounds, often yielding only a few viable candidates. To address this, they created Fit4Function, a library optimized for gene packaging and validated in human cells and mice. Using this data, they built multiple ML models to predict specific capsid functions, ultimately combining them to design multifunctional libraries optimized for several therapeutic traits. Another example is a collaboration between Wyss Institute and Google Research, which applied DL to create highly diverse AAV capsid variants, generating over 57,000 variants with much higher diversity than naturally occurring serotypes. These variants offer potential applications in creating AAV capsid structures before physical testing, including predicting viral assembly using big data from next-generation sequencing (Riyad and Weber, 2021; Boettner, 2021; Eid et al., 2024).

Therefore, AI/ML is showing strides, but issues remain to be addressed. Some significant hurdles still to be overcome are the big data available and the data used to create the ML and DL models. These challenges and regulations in the AI/ML world are constantly controversial. However, with strong suggestions, future research will continue to leverage AI/ML and DL to refine vector design, enhancing targeting specificity and reducing immunogenicity. By incorporating these methodologies, it holds promise to create more efficient, targeted, and immunogenically silent AAV vectors, significantly enhancing the therapeutic potential of gene therapy (Chakraborty et al., 2024).

## Conclusion

The Parvoviridae family, emphasizing AAV vectors, has emerged as a foundation of gene therapy, offering a promising strategy for treating various genetic disorders. Through structural studies, insights into the Parvoviridae family have been gained, clarifying key determinants of tissue tropism and the mechanisms underlying viral infection and transduction. Comparative studies utilizing Cryo-EM and X-ray crystallography have been instrumental in refining our understanding of capsid structures and enhancing AAV vector design and efficacy. These studies have laid the groundwork for developing vectors with improved tissue specificity and reduced immunogenicity.

The combinational use of structural studies and traditional methods, as well as tools such as molecular dynamics simulations and ML algorithms, have enabled the optimization of capsid designs for stability and targeted delivery. Structure-guided evolution, directed evolution, and *in silico* design approaches have provided valuable insights into capsid-receptor interactions, facilitating the creation of AAV vectors with enhanced properties. These advancements have contributed to the development of vectors that can navigate some of the complexities of the human immune system and deliver to specific tissues.

Despite these advancements, challenges such as overcoming immunogenicity and achieving efficient targeted delivery remain. However, incorporating advanced computational methods, including AI/ML and DL, holds a great promise for addressing these challenges. By applying these technologies, future research aims to refine vector design further, enhancing targeting specificity and reducing immune responses. The ongoing advancements in structural bioinformatics, computational modeling, and AI/ML are contributing factors, creating a pathway for more effective and precise treatments for various genetic disorders. Not to mention, through continued innovation and interdisciplinary collaboration, the therapeutic potential of AAV vectors holds great promise.
